# Mechanism and kinetic model of microalgal enzymatic hydrolysis for prospective bioethanol conversion†

**DOI:** 10.1039/d3ra01556d

**Published:** 2023-07-17

**Authors:** Padil, Meilana Dharma Putra, Muslikhin Hidayat, Rina Sri Kasiamdari, Anisa Mutamima, Koji Iwamoto, Muhammad Arif Darmawan, Misri Gozan

**Affiliations:** a Department of Chemical Engineering, Riau University Pekanbaru 28293 Indonesia; b Department of Chemical Engineering, Lambung Mangkurat University Banjarbaru 70713 Indonesia mdputra@ulm.ac.id; c Department of Chemical Engineering, Gadjah Mada University Yogyakarta 55284 Indonesia; d Department of Biology, Gadjah Mada University Yogyakarta 55281 Indonesia; e Department of Environmental Engineering and Green Technology, Universiti Technologi Malaysia Kuala Lumpur 54100 Malaysia; f Research Center for Process and Manufacturing Industry Technology, Research Organization for Energy and Manufacture, National Research and Innovation Agency Jakarta Pusat 10340 Indonesia; g Department of Chemical Engineering, University of Indonesia Depok 16424 Indonesia; h Research Center for Biomass Valorization, University of Indonesia Depok 16424 Indonesia

## Abstract

*Tetraselmis chuii* is a potential microalgae that is in consideration for producing bioethanol owing to its large content of carbohydrates. The glucose production from *T. chuii* through an enzymatic process with cellulase and xylanase (pretreatment process) and α-amylase and glucoamylase (saccharification process) was studied. The mechanism of the enzymatic process was developed and the kinetic models were then evaluated. For the pretreatment process, enzymes with 30% concentration reacted at 30 °C for 40 min resulted in 35.9% glucose yield. For the saccharification process, the highest glucose yield of 90.03% was obtained using simultaneous α-amylase (0.0006%) and glucoamylase (0.01%) enzymes at 55 °C and for 40 min. The kinetic models fitted well with the experimental data. The model also revealed that the saccharification process performed better than the pretreatment process with a higher kinetic constant and lower activation energy. The proposed kinetic model plays an important role in implementing processes at a larger scale.

## Abstract 2

The kinetic model was developed to confirm the enzymatic mechanisms for the pretreatment and the saccharification processes. The proposed kinetic model fitted well with the experimental data.

## Graphical abstract

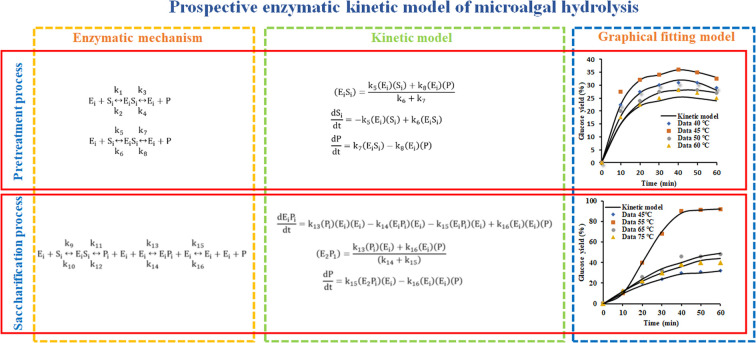

## Introduction

The increasing rate of population has brought some impacts on many aspects of life, such as the increase in the need for vehicles/transportation and dependence on the industry as a supply of human necessities. These consequently give effect to the increase in the demand for fuel.^1^ While, raw material reserves of fossil fuels tend to decrease over time.^2,3^ The excessive use of fossil fuels might also cause environmental pollution and climate change.^4^

Alternative energy sources of bioethanol, mainly derived from agricultural crops containing starch, such as corn and cassava, have been widely explored.^5–7^ However, still there are several problems with using crops as raw materials for bioethanol, including the competition in the use of raw materials between food and energy products.^8^ Second-generation bioethanol derived from lignocellulosic biomass offers a prospective choice owing to its abundant availability and it does not compete directly with food products.^9,10^ However, another problem still exists in relation to the high lignin content that should be removed as it hampers the commercialization of energy products.^11–13^ As such, microalgae, which does not contain lignin, are available as a source of potentially affordable raw material for bioethanol with a fairly fast growth rate and are even able to grow on critical land, waste, and peat water.^14–16^

Microalgae have a high carbohydrate content that can be converted into glucose; this material is then easily converted to bioethanol.^17–20^ Microalgae can also absorb CO_2_ during the photosynthesis process, thereby reducing the concentration of greenhouse gas emissions in the environment.^21^*Tetraselmis chuii* is a green microalgal species with a high carbohydrate content of up to 60%.^22–24^ The process of converting carbohydrates into glucose can be conducted using chemicals or enzymes.^25–27^ The use of chemicals in this process has a number of advantages, such as affordability and fast processing time. However, some disadvantages of using chemicals are related to the formation of furfural and hydroxymethyl furfural (HMF); those are inhibitors in the further fermentation process of glucose into bioethanol. Furthermore, the formed material also could cause corrosion at high temperatures. Alternatively, the acidic materials should be recovered with high-cost equipment.^28^ In contrast, the process of using enzymes, in addition to being able to produce high yields of glucose, can also be carried out at low temperatures without any formation of inhibitors and additional expensive equipment.^29–31^

The process of converting microalgae into glucose uses an enzyme of α-amylase at the initial stage for the formation of dextrin and is then followed by an enzyme of glucoamylase to produce glucose.^32,33^ The main obstacle at this stage is related to the binding of microalgal starch in a rigid cell wall that cannot be in direct contact with the enzyme.^34,35^ Therefore, a pretreatment process using cellulase and xylanase enzymes is required. Also, both enzymes are able to break down the microalgae's cell wall and convert cellulose and hemicellulose inside the cell wall into glucose.^36,37^ In the next stage, the intracellular starch is converted to dextrin by α-amylase and continued into glucose by glucoamylase.^38,39^

The kinetic models are essential to understand an enzymatic process of glucose production using microalgae. This is required for large-scale implementation and operation of the system with the desired standard.^40^ The kinetic prediction of an enzymatic process using cellulase and xylanase enzymes to produce glucose has been presented elsewhere;^41^ however, the Gompertz model used there could not describe the mechanism inside the enzymatic process in microalgae. The enzymatic kinetics of using glucoamylase and α-amylase with the Michaelis–Menten model was also described elsewhere;^42,43^ however, the detailed mechanism with the intermediate step was not considered.

Based on the previous research, the glucose production in the microalgal hydrolysis should be observed in detail based on the steps of the enzymatic process and the types of microalgae. This study aims to optimize the hydrolysis of the microalgae of *T. chuii* using cellulase and xylanase enzymes (as the pretreatment process) and the hydrolysis of starch microalgae using α-amylase and glucoamylase enzymes (as the saccharification process). The reaction kinetic and the model were also developed in detail to study the mechanism of enzymatic reactions; hence, they can be useful for implementation at an industrial scale.

## Method

### Microalgal culture and growth media

The microalgae *Tetraselmis chuii* used in this study were purchased from the Center for Marine Cultivation Development, Lampung Province, Indonesia. The components of microalgae used in this experiment contained 1.07% fat, 19.57% protein, 49.54% hemicellulose, 10.2% cellulose, and 19.62% starch. The cellulase enzyme from *Aspergillus niger* 22178 was purchased from Sigma-Aldrich, Singapore; the enzyme was in the form of white powder with an activity of 0.8 units per mg solid. Every 0.8 units of cellulase could result in 1.0 mol of glucose from the cellulose substrate at pH 4.0–5.0. Meanwhile, the enzyme endo-1,4-β-xylanase from *Trichoderma longibrachiatum* X2629 was purchased from Sigma-Aldrich, Singapore in solid form with an activity of 1.0 units per mg solid. Each 1.0 xylanase unit could result in 1.0 mol of glucose per minute from the xylan substrate at pH 4.5 and a temperature of 30 °C.

### Enzymatic process of microalgae

The process of microalgal pretreatment used two types of enzymes: cellulase (from *Aspergillus niger* 22178) to convert cellulose into glucose and endo-1,4-β-xylanase (from *Trichoderma longibrachiatum* X2629) to convert hemicellulose into glucose. Pretreatment experiments using cellulase and xylanase enzymes were carried out by varying the temperature and enzyme concentration. The saccharification process was conducted using the α-amylase enzyme (to produce dextrin) and glucoamylase enzyme (to produce glucose), both of which were obtained from *Aspergillus oryzae* (with an enzymatic activity of 1.5 units per mg solid) and *Aspergillus niger* (with an enzymatic activity of 30–60 units per mg solid), respectively. The effect of temperature was also observed in this process.

The first experiment of the pretreatment process was carried out using 500 mg microalgae put in a 250 mL Erlenmeyer flask and 100 mL of a buffer solution was added (mixture of sodium acetate and acetic acid) with a pH of 4.5 where this pH value was the optimum pH for cellulase and xylanase enzymes from 4.0 to 5.0. After the microalgae were dissolved, the Erlenmeyer containing the microalgae was placed into a shaker batch for heating up to 40 °C. The enzymes of cellulase and xylanase with the variation of 10%, 20%, or 30% (w/w) were added to the solution by stirring. The pretreatment was carried out for 60 minutes, and samples were taken every 10 minutes. At the end of each pretreatment process, the solution was centrifuged to separate the solution and solids. The pretreatment solution was then heated at 90 °C for 15 minutes using a water bath. The sample was further placed into the freezer at −30 °C to stop the enzyme activity. To observe the effect of the pretreatment temperature, the enzymatic process with the temperature variation of 40 °C, 45 °C, 50 °C, and 60 °C was conducted. For the second experiment of the saccharification process, a similar procedure was followed by utilizing α-amylase and glucoamylase enzymes. The α-amylase concentrations of 0.0002, 0.0006, and 0.001% (w/w) were varied, while the glucoamylase concentrations of 0.002, 0.006, and 0.01% (w/w) were applied. It also included the temperature variation of 45 °C, 55 °C, 65 °C, and 75 °C.

### Experimental analysis

The contents in microalgae, such as glucose, cellulose, hemicellulose, fat, protein, and starch levels, were analyzed. The cellulose and hemicellulose levels were measured using the Chesson-Datta method.^44^ Meanwhile, the analysis of starch content was carried out by acid hydrolysis^45^ and the fat content was evaluated through the gravimetric method.^46^ The protein content analysis was conducted using the Kjeldahl method.^47^ Glucose analysis was carried out using the reducing sugar method of Nelson Somogyi.^48^ For the absorbance of the sample solution, the glucose content was determined using a UV-vis spectrophotometer with *λ* (wavelength) of 540 nm; and then the standard curve was plotted based on the calibration results. Finally, the yield of glucose was calculated based on the following equation:

1 *X* (g L^−1^) = (2.1086 × OD384) + 0.0058 (*R*^2^ = 0.98)

The following equation examined the glucose yield (%):2
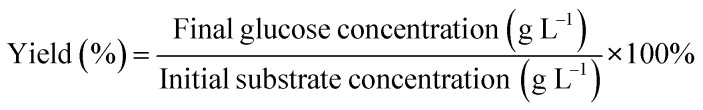


The data were taken in triplicate experiments, and the average data are presented. Statistical analysis was conducted by using ANOVA.

### Mechanism and kinetic model of the enzymatic process

The enzymatic kinetic model that explains the phenomena occurring during the enzymatic process was introduced by Michaelis.^49^ They assumed that the enzyme directly interacts with the substrate stoichiometrically to form the substrate enzyme, leading to thermodynamic equilibrium. This experiment's enzymatic model is a simultaneous process with cellulose and hemicellulose substrates using cellulase and xylanase enzymes. The enzymatic reaction is described by eqn (3) and (4) for cellulase and xylanase enzymes, respectively.3
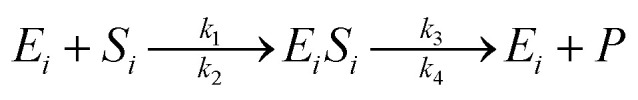
4
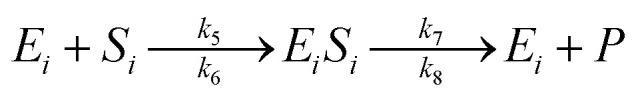


The kinetic model for the intermediate product (*E*_*i*_*S*_*i*_) for eqn (3) is described as follows:5
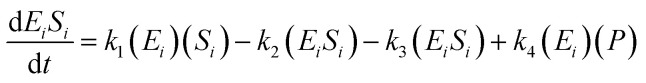


If the pseudo-steady state hypothesis is assumed, 
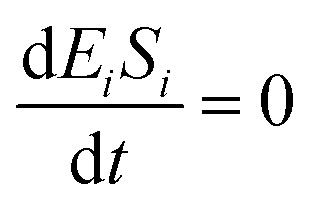
; thus, eqn (5) leads to the following:6
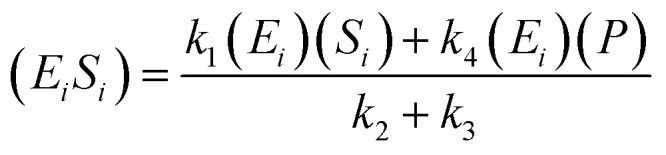


The kinetic model for the substrate (*S*_*i*_) and the product from eqn (3) is described as follows:7
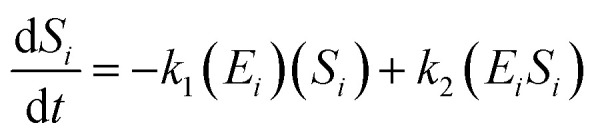
8
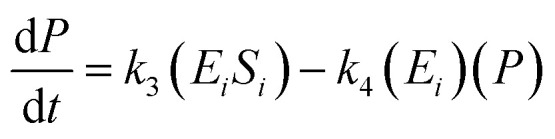


A similar step for eqn (4) was applied, and the following equations were obtained:9
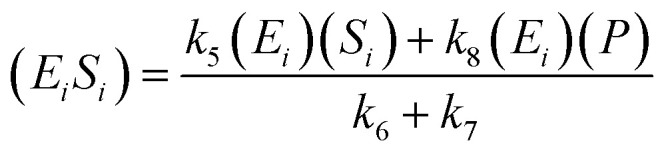
10
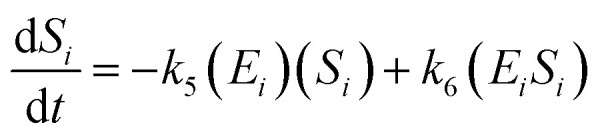
11
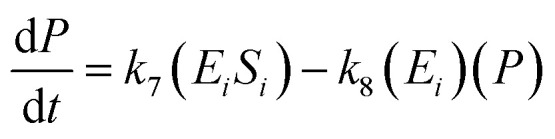


To solve the kinetic model of the enzymatic pretreatment process of cellulose and hemicellulose into glucose, the eqn (6)–(11) were simultaneously applied using MATLAB.

The saccharification process using α-amylase (to produce dextrin) and glucoamylase (to produce glucose) enzymes are described in a model (12):12
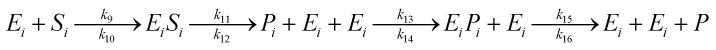


The kinetic model for the first intermediate product (*E*_*i*_*S*_*i*_) is described as follows:13
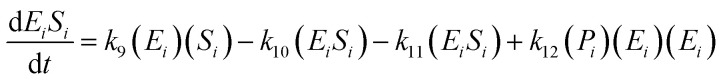


If the pseudo-steady state hypothesis is assumed, 
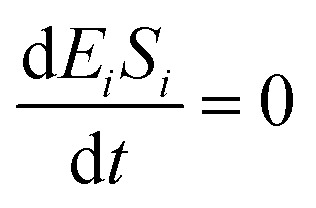
; thus, eqn (13) leads to the following:14
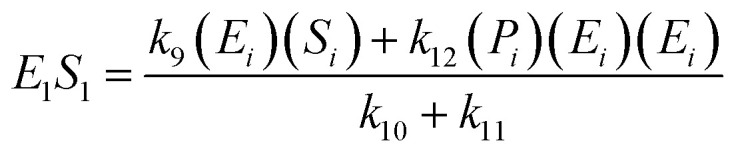


The kinetic model for the substrate (*S*_*i*_) and the product (dextrin) from eqn (12) is described as follows:15
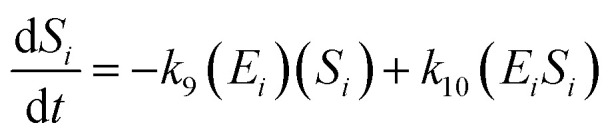
16
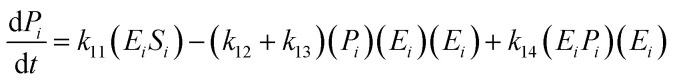


The kinetic model for the second intermediate product (*E*_*i*_*S*_*i*_) is described as follows:17
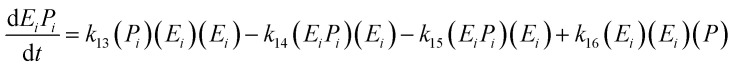


If the pseudo-steady state hypothesis is assumed, 
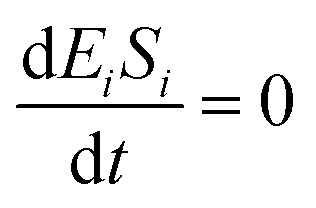
; thus, eqn (17) leads to the following:18
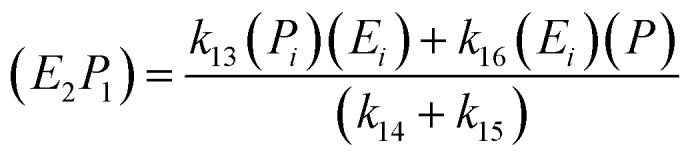


The equation for the final product can be obtained as follows:19
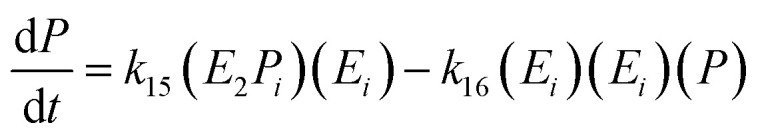


To solve the simultaneous kinetic model of the enzymatic saccharification process of starch into glucose using α-amylase and glucoamylase enzymes, the above equations were simultaneously applied using MATLAB.

To solve the kinetic model of enzymatic pretreatment and the saccharification process above, the ordinary differential equations were solved by using “ODE45” embedded with “fminsearch” to obtain the kinetic constants in MATLAB. The standard error was also examined for each evaluation by the following equation:20
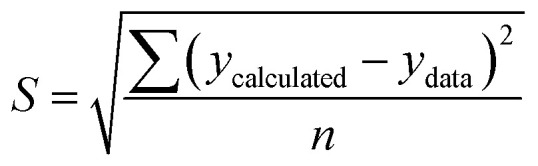


## Results and discussion

### Enzymatic pretreatment process

**Fig. 1:**
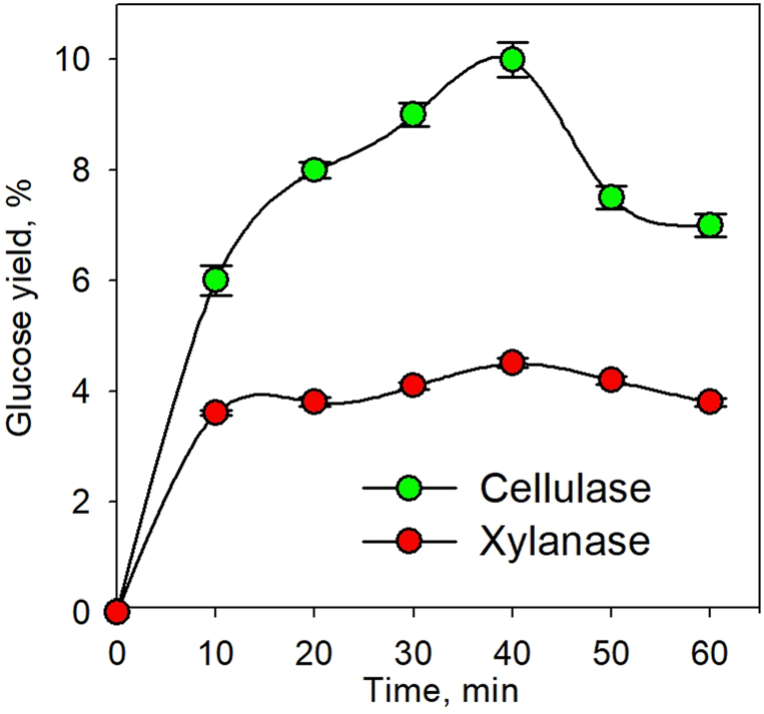
Kinetic profile of glucose yield for the enzymatic pretreatment process using xylanase and cellulase at 45 °C.

Fig. 1 shows the kinetic profile of glucose yield for the pretreatment process using individual xylanase and cellulase enzymes at an enzyme concentration of 10% and temperature of 45 °C. As shown in the figure, the glucose yield increased with increasing time up to 40 min. The increasing time led to greater enzyme activity. This caused more enzymes binding the substrate to form an enzyme–substrate complex; consequently, more products were formed.^50–52^ The decrease in glucose yield observed after 40 min was plausible due to the reduction of the substrate along with time. Conversely, the produced glucose underwent an advanced process in the form of an oxidation process. Glucose was oxidized to form 2 pyruvate with byproducts in the form of 2 NADH and 2 ATP; therefore, as time increased, the pyruvate would also undergo oxidative decarboxylation to form 2 acetyl Co-A.^53^

**Fig. 2:**
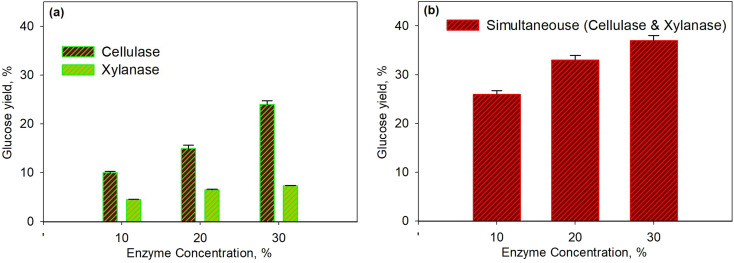
Effect of enzyme concentration on glucose yield for pretreatment process at 45 °C and for 40 min using xylanase and cellulase enzymes: series enzymatic process (a); simultaneous enzymatic process (b).

Fig. 2 shows the effect of enzyme concentration on glucose yield for the pretreatment process at 45 °C within 40 min. The enzymatic process using the xylanase enzyme was continued by the cellulase enzyme as shown in Fig. 2a, while the simultaneous enzymatic process for both enzymes is shown in Fig. 2b. The higher enzyme concentration led to a higher yield of glucose because the greater enzyme concentration increased enzyme activity; hence, more enzymes would bind to the substrate to form an enzyme–substrate complex and continue to form the product. It was observed that the glucose yield for hemicellulose conversion was smaller than the yield for cellulose. This was because the hemicellulose had the largest component, *i.e.*, xylan, which is a polymer of β (1–4) d-xylopiranose (xylose) with β-1,4-glycoside bonds;^54–56^ This then caused the branched xylan chains and led to a more complex structure. Moreover, the hemicellulose acted as a glue in every single cell and induced more difficulties for enzymes to form an enzyme–substrate intermediate system.

For a similar condition, the glucose yield obtained from this study was higher than the yield conducted by Harun and Danquah (29.9%).^57^ They used the cellulase enzyme of *Chlorococum humicola* microalgal hydrolysis with the initial pretreatment process using H_2_SO_4_ to improve the yield. It seems that the use of xylanase enzyme in the pretreatment process is more effective than performing acid hydrolysis. As shown in Fig. 2b, the simultaneous use of enzymes was much better than the use of enzymes separately. This was because each enzyme was specific to selectively attack certain substrates as the xylanase enzymes only break down hemicellulose. Though the hemicellulose is the outer cell wall of microalgae compared to cellulose, the breakdown of cellulose directly after that of hemicellulose was needed; consequently, the simultaneous enzymatic process was more effective.

**Fig. 3:**
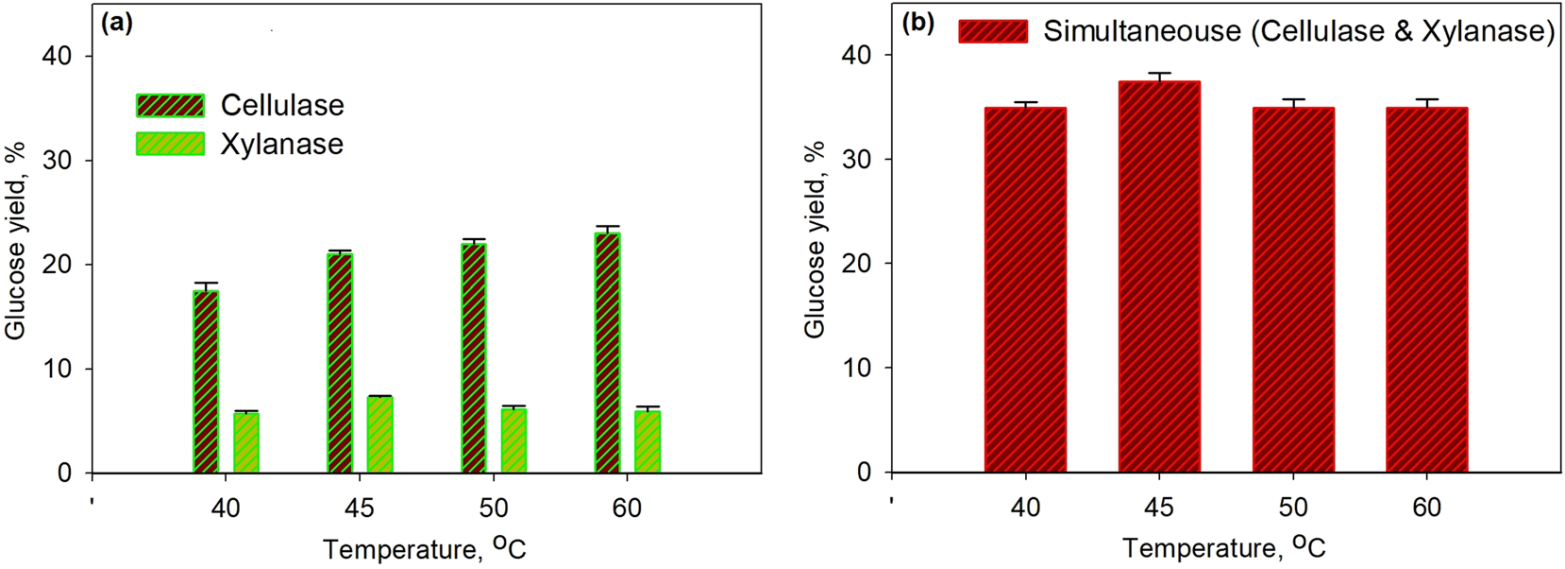
Effect of temperature on glucose yield for the pretreatment process at 30% enzyme concentration and for 40 min using xylanase and cellulose enzymes: series enzymatic process (a); simultaneous enzymatic process (b).

Fig. 3 shows the effect of temperature on glucose yield for the pretreatment process at 30% enzyme concentration for 40 min. The series of enzymatic processes (Fig. 3a) were compared to the simultaneous enzymatic process (Fig. 3b). The glucose yield increased with the increasing temperature as the temperature would affect the molecular kinetic energy of the pretreatment process; thus, leading to the increase in a collision between the substrate and the enzyme molecule. Enzyme activity with the substrate to form the enzyme–substrate complex would increase along with the increase in temperature; therefore, the rate of metabolic processes increased up to the maximum temperature limit. At the conditions above the maximum temperature, the enzyme denaturation process occurred, where the enzyme would no longer function effectively.^58,59^ On the other hand, the produced glucose underwent an oxidation process to form pyruvic acid. It was observed that at a temperature of over 45 °C the glucose yield for the xylanase enzyme dropped though the yield was relatively constant for cellulase. The same finding was observed for simultaneous processes as a 37.5% yield was obtained with the optimum temperature of 45 °C. The optimum temperature obtained from this study is in accordance with the optimum temperature from the other work^58^ as for the cellulase enzyme it is in the range 30–45 °C and for the xylanase enzyme it is 35–50 °C.

### Enzymatic saccharification process

The first step in the enzymatic saccharification of starch in *T. chuii* was the liquefaction of starch using the enzyme α-amylase that broke α-d-(1–4) glycosidic arbitrarily. α-Amylase is an endo enzyme that works to break α-1,4 glycoside bonds randomly on the inside of amylose and amylopectin.^60,61^ The products resulting from the starch saccharification process using the α-amylase enzyme were dextrin and the glucoamylase enzyme hydrolyzes α-d (1–4) and α-d (1–6) glycosidic into glucose.^5,32^

**Fig. 4:**
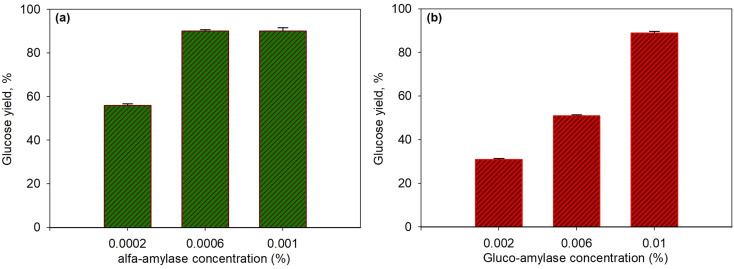
Effect of enzyme concentration on glucose yield for saccharification process at 55 °C and for 40 min using α-amylase and glucoamylase enzymes: various α-amylase with glucoamylase 0.01% (a); various glucoamylase with α-amylase 0.0006% (b).

Both concentration variations were carried out to observe the effect of α-amylase and glucoamylase enzymes on the glucose produced. Fig. 4a and b present the effect of concentration on α-amylase enzymes (with 0.01% glucoamylase) and glucoamylase (with 0.0006% α-amylase) on the saccharification of *T. chuii* starch at 55 °C and for 40 min, respectively. As shown in the figure, at the high concentration of α-amylase enzyme (0.0006%), the glucose yield increased to 90.03%. However, at a higher concentration of 0.001%, the yield produced was relatively constant at 90%. The increase in enzyme activity, which continued to increase only up to a concentration of 0.0006%, was due to the decrease in the available substrate; therefore, enzymes that reacted with substrates formed more stable enzyme–substrate compounds. Consequently, adding a higher amount of enzyme did not further affect the enzyme's activity on the substrate. The resulting dextrin, following this step, was converted into glucose by glucoamylase at a constant concentration. As a result, the effect of glucoamylase was relatively the same in this case.

Likewise, in the variation of glucoamylase, as shown in Fig. 4b, when dextrin was formed by α-amylase under constant concentration conditions, increasing the concentration of glucoamylase from 0.002% to 0.01% significantly increased the yield of glucose from 31% to 90.3%. This indicated that the enzymatic process in the second stage was crucial because quite a lot of dextrin formed must be immediately converted into glucose with the appropriate amount of enzyme is required. If not immediately converted, dextrin would react back to produce complex substrate–enzyme components.

The results of this study are also in line with the research conducted by Choi *et al.*^32^ showing that the concentration of α-amylase and glucoamylase enzymes greatly affected the amount of the resulting product. However, they used the enzymes α-amylase and glucoamylase separately in each experiment with dextrin and glucose analysis, respectively. This research showed that the optimum concentration of the enzyme α-amylase was 0.005% at the temperature of 90 °C within 30 min. In comparison, the optimum glucoamylase was 0.2% at a temperature of 55 °C for 30 min as only 23% glucose yield was produced. Although the levels of the glucoamylase enzyme were much higher than those in our work, and the substrate was pure dextrin (no way to form a starch-α-amylase complex), the glucose was still low. The presence of α-amylase that would form a complex compound (starch-α-amylase) helped in controlling the subsequent reaction between glucoamylase and dextrin to produce glucose. Hence, the presence of dextrin, which is not too high, can be converted quickly into glucose.

**Fig. 5:**
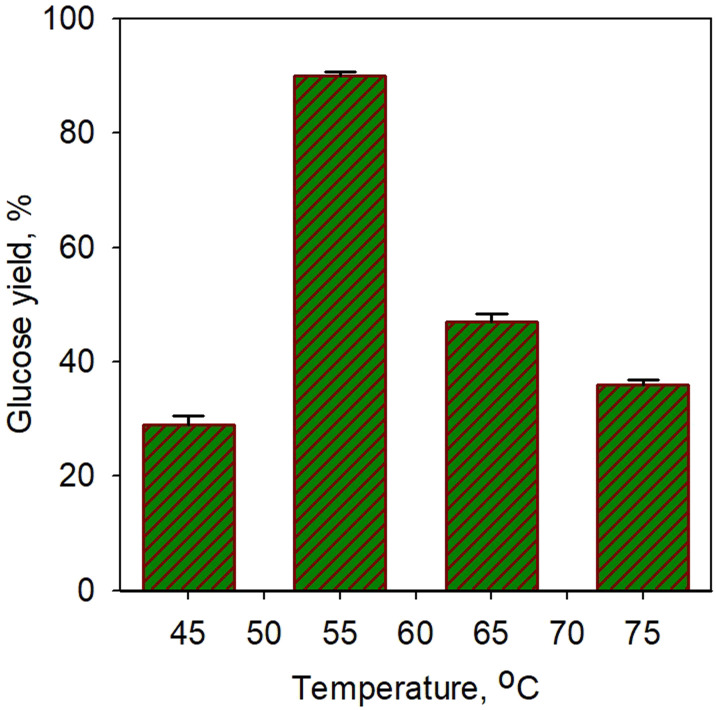
Effect of temperature on glucose yield for saccharification process using simultaneous α-amylase (0.0006%) and glucoamylase (0.01%) enzymes for 40 min.

Fig. 5 shows the effect of temperature on glucose yield for the saccharification process using simultaneous α-amylase (0.0006%) and glucoamylase (0.01%) enzymes for 40 min. A sharp increase in glucose yield (3-fold) was obtained from 29% at 45 °C to 90.3% at 55 °C. However, a significant drop in yield was observed at temperature afterwards. The increase in enzyme activity towards temperature should increase metabolic processes due to the increased kinetic energy of the reacting molecules, but only up to the optimal temperature limit. A very extreme denaturation process of the enzyme occurred;^62^ a drop in yield to 47% was obtained at a temperature of 65 °C. However, the glucose yield obtained in our study was found to be higher than that of Megawati *et al.*^63^ In their work, 80% glucose yield was obtained using α-amylase and glucoamylase at a relatively high temperature of 80 °C for 5 h; thus, it is certainly less economical.

Table 1 presents the hydrolysis yield for various microalgae, enzymes, and operating conditions. The research using individual enzymes showed that the biomass source of *Chlamydomonas reinhardtii* using the α-amylase enzyme produced the highest yield of 64.2%.^32^ Meanwhile, the experiment results of Megawati *et al.*^63^ using simultaneous α-amylase and glucoamylase enzymes were better than those carried out by Phwan *et al.*^64^ with quite high yields, *i.e.*, 80% compared to 35%; even though they used the same microalgal source, *i.e.* Chlorella. This may be due to the use of 50 °C compared to 32 °C. As reported, the optimum temperature for using α-amylase and glucoamylase enzymes is in the range of 50–55 °C to 55–60 °C, respectively. However, our results showed a higher yield of 90%; this could be due to the type of microalgae used, thus affecting the production of glucose.

**Table 1:** Comparison data of glucose production from several biomass and microalgae

Biomass source	Enzymes	Operation conditions	Hydrolysis yield	Reference
*Tetraselmis chuii*	α-Amylase + glucoamylase	pH 4.5, *T* = 55 °C	90.03%	This research
*Chlorella*	α-Amylase + glucoamylase	pH 5, *T* = 50 °C	80%	63
*Chlorococum humicola*	Cellulase	pH 4.8, *T* = 40 °C	29.9%	57
*Chlamydomonas reinhardtii*	α-Amylase	pH 5, *T* = 55 °C	64.2%	32
Mixed microalgae	Cellulase	pH 5, *T* = 50 °C	57%	65
Mixed microalgae	Cellulase	pH 4.6, *T* = 50 °C	62%	66
*Chlorella*	α-Amylase + glucoamylase	pH 5.5, *T* = 32 °C	35%	64

### Statistical analysis

Statistically, in the pretreatment process, the effect of the enzyme concentration for each parallel process and simultaneous process on the glucose yield was insignificantly different at *p* < 0.05. However, the interaction for both processes was statistically different at *p* < 0.05. The same trends were observed for the effect of temperature for both processes on glucose yield. For the saccharification process with the effect of enzyme concentration, the use of α-amylase resulted in a significant difference at *p* < 0.05 and the use of glucoamylase was, however, statistically insignificant at *p* < 0.05. Furthermore, the interaction between both enzymes showed a statistically significant impact at *p* < 0.05. A similar observation was seen for the effect of temperature on glucose yield for the saccharification process.

### Enzymatic saccharification process

**Fig. 6:**
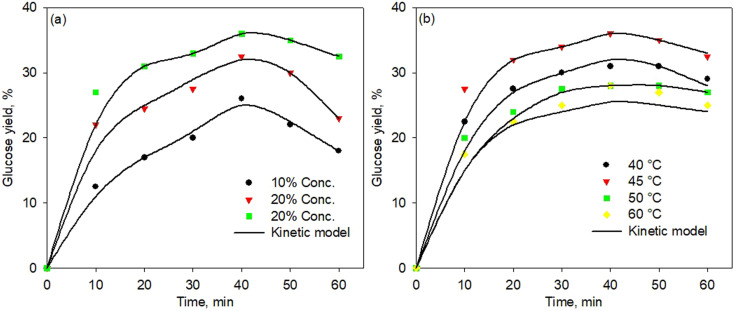
Kinetic model of simultaneous cellulose and hemicellulose pretreatment process at various temperatures (a) and enzyme concentration (b).

Fig. 6a and b show the kinetic model of the enzymatic pretreatment process using simultaneous cellulose and hemicellulose at various temperatures and enzyme concentrations, respectively. The model well fitted the experimental data for all temperatures and concentrations. Although the fitting points were less precise at a high temperature of 60 °C, the standard error was found still low, *i.e.*, 0.0228. Thus, the proposed kinetic model was developed very well for all temperatures and concentrations with a standard error below 0.03.

Table 2 shows the data for kinetic constants of the enzymatic pretreatment process using simultaneous cellulose and hemicellulose at various concentrations. The kinetic constant values for the reaction presented in the table correspond to the kinetic model as shown in Fig. 6. As observed in Table 2, the higher enzyme concentration resulted in a greater rate constant, thus increasing glucose production. The reaction rate constants *k*_1_ and *k*_5_ play an important role in reducing cellulose and hemicellulose substrates, respectively, by producing intermediate enzyme–substrate (ES) products. The greater value of *k*_1_ and *k*_5_ led to the faster reduction of cellulose and hemicellulose concentration to produce the intermediate products. On the other hand, the reaction rate constants of *k*_3_ and *k*_7_ played a significant role in changing intermediate compounds to produce glucose. It means that the higher values of *k*_3_ and *k*_7_ contributed to the faster decrease in the intermediate (ES) component to produce glucose.

**Table 2:** Kinetic constants for simultaneous cellulose and hemicellulose pretreatment process at various enzyme concentrations

Enzyme concentration% (w/w)	Reaction rate constant (min^−1^)								Standard error
	*k*_1_	*k*_2_	*k*_3_	*k*_4_	*k*_5_	*k*_6_	*k*_7_	*k*_8_	
10	0.44	0.01	0.01	0.06	0.19	0.01	0.08	0.21	0.0291
20	0.64	0.19	0.11	0.08	0.26	0.014	0.16	0.37	0.0125
30	1.11	0.28	0.12	0.32	0.32	0.042	0.44	0.38	0.0210

It was also observed that the value of *k*_1_ was higher than *k*_5,_ indicating that the cellulose conversion rate into intermediates was faster than the rate of hemicellulose into that one. However, the conversion rate of xylanase to convert hemicellulose into an intermediate became crucial due to the higher content of hemicellulose (49.54%) than that of cellulose (10.2%). Based on the data in Table 2, the values of *k*_1_ and *k*_5_ were found smaller than the values of *k*_3_ and *k*_7_; indicating that the rate of glucose production was slower than the rate of formation of intermediate compounds (ES). Therefore, this confirmed that glucose production became the determining step for the reaction rate as the slowest step in the kinetic model was observed.^67–69^

Table 3 shows the data for kinetic constants of the enzymatic pretreatment process using simultaneous cellulose and hemicellulose at various temperatures. As shown in the table, the value of *k* increased with increasing temperature, but the kinetic constant decreased at 50 °C and even more at 60 °C. This finding confirmed the previous results that were related to the denaturation process occurring at a temperature of 50 °C for both cellulase and xylanase enzymes, thereby reducing the effectiveness of the enzyme. As reported, the cellulase enzyme has an optimum range temperature of 45–50 °C, and the denaturation process occurred at a later temperature;^62,70^ while the xylanase enzyme had an optimum range temperature of 50–55 °C and further higher temperature confirmed the denaturation process.^71,72^ However, as shown in Table 3, the *k*_5_ value, relatively, did not have significant changes at all temperatures, and this indicated that the conversion process of hemicellulose substrates into intermediate compounds (ES) proceeded well, and the process did not experience any denaturation. Another possibility is that the increase in temperature impacted the conversion process of intermediate compounds from hemicellulose enzymes to glucose. In addition, the finding data was differently observed for the kinetic cellulase rate as the all-kinetic constants decreased, thus indicating the denaturation effect to be influential on the cellulase enzyme.

**Table 3:** Kinetic constants for simultaneous cellulose and hemicellulose pretreatment process at various temperatures

Temperature (°C)	Reaction rate constant (min^−1^)								Standard error
	*k*_1_	*k*_2_	*k*_3_	*k*_4_	*k*_5_	*k*_6_	*k*_7_	*k*_8_	
40	0.20	0.12	0.004	0.21	0.27	0.002	0.37	0.27	0.0149
45	1.11	0.28	0.12	0.32	0.32	0.04	0.44	0.38	0.0210
50	0.03	0.25	0.11	0.12	0.32	0.01	0.03	0.011	0.0223
60	0.001	0.02	0.002	0.09	0.29	0.001	0.01	0.001	0.0228

**Fig. 7:**
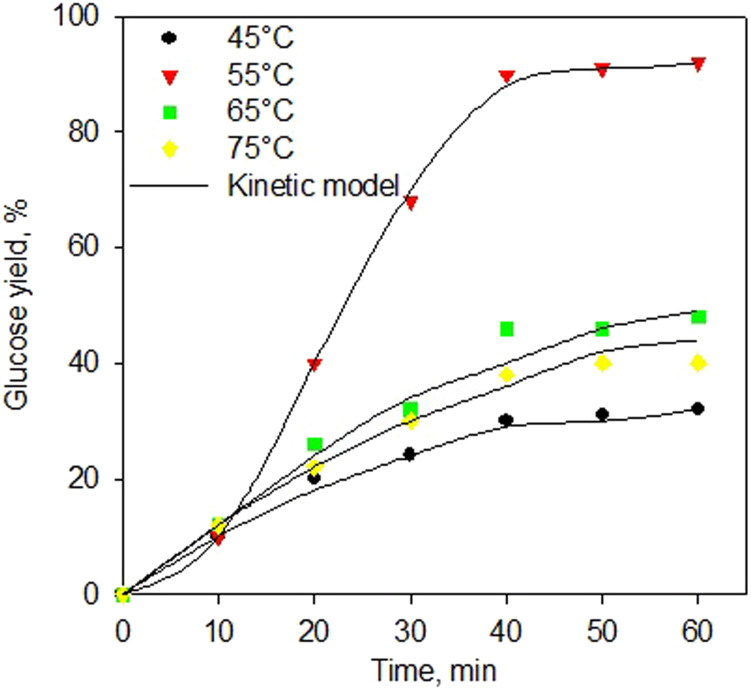
Kinetic model of saccharification process using simultaneous α-amylase and glucoamylase enzymes at various temperatures.

Fig. 7 shows the kinetic model of the enzymatic saccharification process using α-amylase and glucoamylase enzymes at various temperatures. The kinetic model excellently fitted the experimental data for all temperatures. Again, the proposed kinetic model for the enzymatic saccharification process has been generated very well with a standard error below 0.05. This kinetic development is useful for the implementation process at industrial scale. Table 4 shows the kinetic constants of the enzymatic saccharification process using simultaneous α-amylase and glucoamylase enzymes. As shown in Table 4, the greatest kinetic constant was obtained at 55 °C. The higher temperature should increase the reaction rate constant, but at 65 °C, the reaction rate constants decreased, indicating that the enzyme started to experience denaturation. As reported,^73–75^ the α-amylase enzyme has an optimum range temperature of 50–55 °C, and the denaturation process occurs at a temperature of 70 °C. Meanwhile, glucoamylase enzyme has an optimum range temperature of 55–60 °C, and the enzyme with the process at a temperature of 70 °C was observed to be denaturation.^76–78^

**Table 4:** Kinetic constants for saccharification process using simultaneous α-amylase and glucoamylase enzymes at various temperatures

Temperature (°C)	Reaction rate constant (min^−1^)								Standard error
	*k*_9_	*k*_10_	*k*_11_	*k*_12_	*k*_13_	*k*_14_	*k*_15_	*k*_16_	
45	0.09	0.02	1.23	0.26	0.30	0.03	0.25	0.14	0.0495
55	0.14	0.02	1.45	0.46	0.40	0.27	0.50	0.25	0.0073
65	0.09	0.02	1.37	0.34	0.35	0.20	0.21	0.18	0.0278
75	0.06	0.01	1.29	0.22	0.10	0.03	0.19	0.14	0.0259

As presented in Table 4, the value of *k*_11_ was higher than that of *k*_9_, indicating that the process of forming dextrin (P1) was more influential than the conversion of starch into a complex compound or intermediate 1 (ES1). The value of *k*_13_ was greater than that of *k*_9_, indicating that the conversion rate of dextrin to intermediate 1 (ES1) was more dominant than the rate of conversion of starch to intermediate 2 (ES2). However, the *k*_14_ value was also quite large, which indicated that the reversible rate of intermediate 2 into dextrin was also quite significant. On the other hand, the higher value of *k*_11_ compared to the *k*_15_ indicated that the dextrin production rate was higher than the glucose production rate from each intermediate (ES1 and ES2, respectively). This might be because the reaction was simultaneous; hence, the rapid production of dextrin was directly bound by the glucoamylase enzyme to be converted into glucose, even though it went through the stages of the dextrin-glucoamylase (ES2) complex compound. It then confirmed that the rate of glucose production is the determining step of the reaction rate.

In this study, the effect of temperature was also observed using the Arrhenius equation. The calculated parameter was the activation energy (*E*_a_), as shown in Table 5. The activation energy for the series of pretreatment processes from cellulose to glucose followed by hemicellulose to glucose had a higher value (31.8 and 30.37 kJ mol^−1^) compared to the one for the simultaneous pretreatment process (18.21 kJ mol^−1^). This indicated that the reaction rate of glucose formation in the simultaneous process was faster than in the series process, as a larger value of *k* was observed. Also, the glucose production for the simultaneous pretreatment process from cellulose and hemicellulose had a higher activation energy value (18.21 kJ mol^−1^) than the production for the simultaneous saccharification process (11.29 kJ mol^−1^). Thus, the reaction rate for the saccharification process was also faster than the rate for the pretreatment process, as indicated by the greater *k* value. This study's results are aligned with the research conducted by Harun and Danquah^57^ in which a smaller activation energy led to a faster reaction speed.

**Table 5:** Arrhenius parameter, activation energy, and rate constant of pretreatment process and saccharification reaction

Product	Substrate and enzyme	Enzyme	*E*_a_ (kJ mol^−1^)	*k*_7_/*k*_15_/*k*_p_ (min^−1^)
**Pretreatment process (*T* = 45 °C); pH = 4.5; enzyme: 30% (w/w)**				
Glucose	Cellulose	Cellulase	31.80	0.21
Glucose	Hemicellulose	Xylanase	30.37	0.09
Glucose	Cellulose and hemicellulose	Cellulase and xylanase	18.21	0.44

**Saccharification process (*T* = 55 °C); pH = 4.5; enzyme: α-amylase of 0.0006% (w/w) and glucoamylase 0.01% (w/w)**				
Glucose	Starch	α-amylase and glucoamylase	11.29	0.50

As shown in Table 5, the value of *E*_a_ in the pretreatment process was small, while the value of *k* became larger at higher temperatures. This is according to the Arrhenius equation 
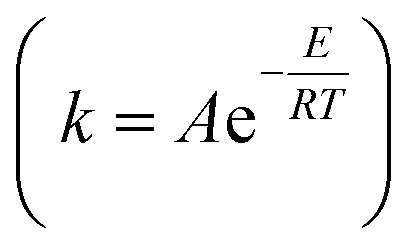
 that the higher temperature leads to a smaller negative exponent value in the Arrhenius equation; hence, the greater value of the reaction rate constant (*k*) is obtained. This consequences in a faster reaction rate. If the temperature of the pretreatment process is too high, it will cause the enzyme to deactivate more quickly. As previously observed in this study, the value of the reaction rate constant decreased at temperatures above 45 °C. In addition, the use of simultaneous enzymes and the increase in the concentration of enzymes led to a smaller value of *E*_a_, thus resulting in a larger value of *k*. It was reasonable because when more enzymes are used, more possibility for the substrate to form an enzyme–substrate complex, thereby accelerating the reaction. As reported,^79^ an increase in the enzyme in the substrate increased to the dextrose equivalent, indicating a faster reaction.

## Conclusions

The pretreatment process using cellulase and xylanase enzymes and the saccharification processes using α-amylase and glucoamylase enzymes were successfully carried out to produce high glucose yield from *Tetraselmis chuii* microalgae. Increasing enzyme concentration increased the glucose yield, while the yield increased with the temperature. The enzymatic hydrolysis of the pretreatment process with an enzyme concentration of 30% at 30 °C resulted in a glucose yield of 35.9%. The hydrolysis reaction of the saccharification process conducted at 55 °C and pH of 4.5 resulted in a glucose yield of 90.03%. The proposed kinetic model well fitted with the experimental data for both pretreatment and saccharification processes. The kinetic studies revealed that in the pretreatment process, the denaturation process was observed at the conversion of the intermediate compounds from hemicellulose enzymes to glucose. Furthermore, in the saccharification process, the rate of glucose production became the determining step of the reaction rate as the dextrin formation rate and intermediate process underwent rapidly. The faster rate of the saccharification process was observed compared to the rate of the pretreatment process as indicated by the activation energy of 11.29 kJ mol^−1^ and 18.21 kJ mol^−1^, respectively. The kinetic study showed that the developed kinetic model could be a potential base for implementation at the industrial scale.

## Conflicts of interest

There are no conflicts to declare.

## Supplementary Material
